# Evaluation of saturated and unsaturated fat with vitamin A or beta-carotene supplementation in nursery pigs

**DOI:** 10.1093/tas/txad089

**Published:** 2023-07-28

**Authors:** Sarah Elefson, Laura Greiner

**Affiliations:** Department of Animal Science, Iowa State University, Ames, IA 50011, USA; Department of Animal Science, Iowa State University, Ames, IA 50011, USA

**Keywords:** beta-carotene, fat, nursery, vitamin A

## Abstract

One hundred and fifty-two nursery pigs (PIC, Hendersonville, TN) were randomly assigned to mix sex pens and one of six dietary treatments in a 3 × 2 factorial. Diets included no added fat, 3% added choice white grease, or 3% added soy oil with either a supplemented vitamin A (for a total of 11,640 IU vitamin A/kg, Rovimix A 1000, DSM, Parsippany, NJ, US) or beta-carotene (for a total of 8,708 IU vitamin A/kg equivalent, Rovimix β-Carotene 10%, DSM). Pigs were given a 3-d adaptation period upon arrival. Pigs were weighed at the start of the study and at the end of each phase. A blood sample was taken from one pig per pen at the start and end of the study. Tissues were collected from eight pigs at the start of the study and six pigs per treatment at the end of the study. Data were analyzed via the GLIMMIX procedure in SAS 9.4 (SAS Inst., Cary, NC). Pen was the experimental unit, and repeated measures were used for growth performance and blood parameters. There was no fat by supplement interaction (*P* > 0.05) on body weight (**BW**), but there was a tendency (*P* = 0.054) for heavier BWs when soy oil was added to diets. There was no difference (*P* > 0.05) in average daily feed intake or average daily gain (**ADG**). There was an improved gain:feed (*P* = 0.02) when pigs were fed choice white grease over no added fat. There were time differences (*P* < 0.05) for plasma vitamins A (retinol), D (25 hydroxy vitamin D3), and E (alpha-tocopherol). Vitamin A and D values were higher at the end of the study, whereas vitamin E values were lower at the end of the study. The choice white grease diets had the highest (*P* < 0.05) plasma vitamins D and E (6.74 ng/mL and 2.87 ppm, respectively). Pigs supplemented with vitamin A had higher (*P* < 0.05) hepatic vitamin A than pigs supplemented with beta-carotene (19.9 vs. 15.6 ppm, respectively). There were no differences (*P* < 0.05) between immunoglobulins G and M or mRNA abundance of select genes (retinol binding protein 2, alcohol dehydrogenase class 1, lecithin retinol acyltransferase phosphatidylcholine-retinol O-acyltransferase, and beta-carotene oxygenase 1). In conclusion, fat inclusion level and type, with either vitamin A or beta-carotene supplementation, did not affect the overall nursery pig growth performance. The addition of fat resulted in an increase in ADG and BW. Diets with choice white grease had the highest plasma vitamins D and E, and supplemental vitamin A increased hepatic vitamin A.

## Introduction

Vitamins A, D, and E are all fat-soluble vitamins (Combs, 2012) and have critical roles in the body. Vitamin A is responsible for vision, epithelium cell differentiation, and supporting the innate immune system (Huang et al., 2018). Vitamin D has a role in the immune system as many immune cells have vitamin D receptors (Aranow, 2011). Vitamin E is also important in supporting immune function (Lewis et al., 2019) and acts as a strong antioxidant (Combs, 2012). Fat-soluble vitamins have similar features in their structures, such as being comprised of a five-carbon isoprenoid derived initially from acetyl CoA (Combs, 2012). Fat-soluble vitamins are absorbed in the small intestine in the presence of fat (Albahrani and Greaves, 2016) and via the same pathways in which lipids are absorbed (Iqbal and Hussain, 2009). Fat-soluble vitamins are encompassed in micelles and then incorporated into chylomicrons for absorption in the upper half of the small intestine (Iqbal and Hussain, 2009). Fat is known to be absorbed in the upper half of the small intestine; thus, fat-soluble vitamins are believed to be absorbed in the same location as fat (Reboul, 2013b).

There are two main classifications of fat: saturated and unsaturated. Saturated fats tend to be of animal origin, while unsaturated fats tend to be of vegetable origin (Mani and Kurpad, 2016). Saturated fats are typically solid at room temperature and include choice white grease and beef tallow (Stahly, 1996). Unsaturated fats are normally liquid at room temperature and include vegetable oils such as corn and soy (Stahly, 1996). The digestibility of fats can be determined by their unsaturated to saturated fat ratio, with digestibility increasing with a lower unsaturated to saturated ratio (Powles et al., 1995). Typically, saturated fats tend to be harder to digest as they have a lower unsaturated to saturated ratio (Powles et al., 1995), due to the lack of double bonds in saturated fats that are found in unsaturated fats. Since saturated fats tend to be harder to digest, there is the potential for it to be more difficult to absorb fat-soluble dietary components, such as fat-soluble vitamins, that are absorbed with fats in the small intestine.

General absorption factors that affect the intestinal absorption of fat-soluble vitamins were summarized by Borel (2003), in which the presence of any fat in the diet increases the absorption of the fat-soluble vitamins. The previous has also been observed in swine models, focused specifically on vitamin E, and concluded that diets with added saturated fats (choice white grease and beef tallow) have an increase fat-soluble vitamin E in circulation at the end of the study (Moreira and Mahan, 2002; Lauridsen, 2010). There is limited data that overall looks at the effect of added dietary fats and fat-soluble vitamins in young pigs, as historically fat in young pig diets is added for palatability (Miller et al., 1991). There is even less work that explores vitamin A supplementation in young pigs, as it is a more regulated fat-soluble vitamin. The lack of work that investigates how different fat types influence fat-soluble vitamin absorption in young nursery pigs led to the objective of this study. The objective of this study is to assess the effects of two common swine dietary fat sources, with the supplementation of vitamin A or beta-carotene on fat-soluble vitamins in circulation in nursery pigs and their subsequent growth performance.

## Materials and Methods

### Animal Care and Use

All procedures in this experiment adhered to guidelines for the ethical and humane use of animals for research and were approved by the Institutional Animal Care and Use Committee at Iowa State University (IACUC #20-086).

### Animals, Housing, and Management

Nursery pigs (*n* = 152; 5.81 ± 0.01 kg body weight (**BW**); PIC 337 × 1050, PIC, Hendersonville, TN) were randomly assigned to mixed sex pens (*n* = 3 pigs per pen) and allowed a 3-day adaptation period in the nursery facility after weaning. All pigs received a common diet during the adaptation period. After the adaptation period, pens were assigned to dietary treatments. Eight pigs were randomly selected for euthanasia and were not assigned to a dietary treatment. Each pen (96.5 × 182.9 cm) had a single-hole feeder (38 × 30.5 cm feeder space), a water nipple, and slatted floors. The pigs were porcine reproductive and respiratory syndrome virus negative upon arrival and were vaccinated against *Mycoplasma hyopneumoniae* and porcine circovirus prior to weaning.

### Dietary Treatments

Pens were assigned to one of six diets (*n* = 8 pens per diet) in a 3 × 2 factorial that consisted of no added fat, 3% choice white grease (saturated fat source), or 3% soy oil (unsaturated fat source) and supplemented with vitamin A in the form of retinyl acetate (4 ppm for a total of 11,656 IU of vitamin A/kg; Rovimix A 1000; DSM, Parsippany, NJ, US [VA]) or beta-carotene (40 ppm for a total of 8,708 IU of vitamin A/kg after bioconversions, Rovimix β-Carotene 10%; DSM [BC]). The beta-carotene was supplemented with the assumption that 26,700,000 IU vitamin A equivalent/kg of product to obtain 8,708 IU vitamin A/kg of complete feed (NRC, 2012). Beta-carotene was also supplemented to have the same amount of active ingredient as vitamin A. Supplementation of vitamin A and beta-carotene was done in addition to the amount of vitamin A included in the diet premix, which was supplied by the same company as the supplements (DSM). Fat was added at the expense of corn. Diets were not isocaloric. However, the diets with added fat were similar in metabolizable energy (19 kcal/kg difference in metabolizable energy) in all phases. Pigs were given ad libitum access to feed and water. Pen and feeder weights were obtained at the start of the study (day 0), the end of phase 1 (day 7), the end of phase 2 (day 21), and the end of phase 3 (day 40). Diets were nutritionally adjusted during each phase to meet or exceed the pig’s nutritional requirements (NRC, 2012, Table 1).

**Table 1.** Dietary composition formulation and calculations across three nursery phases to ensure all diets meet or exceed NRC (2012) requirements

	Phase 1			Phase 2			Phase 3		
	0% Fat	3% SatFat^1^	3% UnsatFat^2^	0% Fat	3% SatFat	3% UnsatFat	0% Fat	3% SatFat	3% UnsatFat
Ingredient, %									
Corn	36.27	33.07	33.08	45.32	42.32	42.32	61.58	58.58	58.58
Soybean meal 47.5% crude protein	20.00	20.00	20.00	20.00	20.00	20.00	34.00	34.00	34.00
Oat groats	15.00	15.00	15.00	15.00	15.00	15.00	—	—	—
DairLac 80^3^	12.91	12.91	12.91	5.41	5.41	5.41	—	—	—
Fish meal	5.00	5.00	5.00	5.00	5.00	5.00	—	—	—
Plasma protein	3.25	3.25	3.25	2.50	2.50	2.50	—	—	—
Soybean oil	—	—	3.00	—	—	3.00	—	—	3.00
Choice white grease	—	3.00	—	—	3.00	—	—	3.00	—
Dried whey	2.50	2.50	2.50	2.50	2.50	2.50	—	—	—
Monocalcium phosphate	1.83	1.83	1.83	1.17	1.17	1.17	1.41	1.41	1.41
Calcium carbonate	0.90	0.90	0.90	0.78	0.78	0.78	1.07	1.07	1.07
l-Lysine HCL	0.50	5.00	0.50	0.50	0.50	0.50	0.45	0.45	0.45
Zinc oxide	0.38	0.38	0.38	0.38	0.38	0.38	0.00	0.00	0.00
Sodium chloride	0.30	0.30	0.30	0.30	0.30	0.30	0.50	0.50	0.50
Dl methionine	0.29	0.29	0.29	0.27	0.27	0.27	0.25	0.25	0.25
Vitamin premix^4^	0.25	0.25	0.25	0.25	0.25	0.25	0.25	0.25	0.25
l-Threonine	0.21	0.21	0.21	0.20	0.20	0.20	0.18	0.18	0.18
Trace mineral premix^4^	0.15	0.15	0.15	0.15	0.15	0.15	0.15	0.15	0.15
l-Valine	0.34	0.34	0.34	0.14	0.14	0.14	0.12	0.12	0.12
l-Tryptophan	0.08	0.08	0.08	0.07	0.07	0.07	0.04	0.04	0.04
Copper sulfate	0.07	0.07	0.07	0.07	0.07	0.07	—	—	—
Vitamin A^5^	0.0004	0.0004	0.0004	0.0004	0.0004	0.0004	0.0004	0.0004	0.0004
Beta-carotene^6^	0.004	0.004	0.004	0.004	0.004	0.004	0.004	0.004	0.004
Calculated composition									
Metabolizable energy, kcal/kg	3129	3270	3289	3152	3288	3307	3299	3435	3453
Crude protein, %	21.59	21.517	21.516	21.36	21.139	21.139	21.223	21.002	21.002
SID^7^ Lys, %	1.464	1.459	1.459	1.428	1.423	1.413	1.335	1.33	1.33
Calcium, %	1.05	1.049	1.049	0.876	0.875	0.875	0.779	0.777	0.777
Available phosphorous, %	0.71	0.709	0.709	0.521	0.52	0.52	0.371	0.37	0.37

^1^Saturated Fat, added at the expense of corn.

^2^Unsaturated Fat, added at the expense of corn.

^3^DairyLac 80 is a granular, high lactose ingredient manufactured through the process of dry rolling liquid whey permeate that contains 3% crude protein and 80% lactose.

^4^Vitamin and trace mineral premix: Provided 7,656 IU vitamin A, 875 IU vitamin D, 63 IU vitamin E, 3.8 mg vitamin K, 70 mg niacin, 33.8 mg pantothenic acid, 13.8 mg riboflavin, 0.06 mg vitamin B12, 165 mg Zn (zinc sulfate), 165 mg Fe (iron sulfate), 39 mg Mn (manganese sulfate), 16.5 mg Cu (copper sulfate), 0.3 mg I (calcium iodate), and 0.3 mg Se (sodium selenite) per kilogram of diet.

^5^Vitamin A in the form of retinyl acetate supplemented an additional 4 ppm for a total of 11,640 IU of vitamin A/kg complete feed; Rovimix A 1000; DSM, added at the expense of corn.

^6^Beta-carotene supplemented an additional 40 ppm for a total of 8,708 IU of vitamin A/kg complete feed, ROVIMIX β-Carotene 10%; DSM, added at the expense of corn.

^7^Standard ileal digestibility.

### Sample Collection

At the start of the study, an initial blood sample was collected in a dipotassium ethylene diamine tetra acetic acid (K2EDTA) blood tube (7 mL; BD Vacutainer, Franklin Lankes, NJ) via sterile, jugular venipuncture from one pig per pen to establish a baseline of vitamin levels in circulation. Blood samples were centrifuged (Sorvall Legend XFR, ThermoFisher Scientific, Waltham, MA, USA) at 200 × *g* for 10 min at 4 °C to separate the plasma. Furthermore, eight pigs were euthanized via captive bolt followed by jugular exsanguination. Liver (entire right lobe) and jejunum samples were collected to establish a baseline of vitamin concentration in the liver and mRNA abundance in the intestinal samples. Jejunum samples were obtained by measuring 10% of the total intestine length (Elefson et al., 2021) from the duodenum. Roughly 15.24 cm of the intestine was collected, washed with a phosphate buffer saline (PBS; pH 7.4; room temperature), snap-frozen in liquid nitrogen, and stored at −80 °C until further analysis. The right lobe of the liver was collected in a sterile Whirl-Pak bag (Madison, WI) for vitamin analysis. Plasma and liver samples were also stored at −80 °C until further analysis.

At the end of the study, a blood sample (K2EDTA blood tube, 7 mL; BD Vacutainer) was taken via sterile, jugular venipuncture from the same pig sampled at the beginning of the study. A total of six pigs (*n* = 3 barrows and gilts) from each diet were randomly chosen for euthanasia via captive bolt followed by exsanguination. The previously described tissue samples were collected and stored in the same manner. All liver and plasma samples were sent to the Iowa State Veterinarian Diagnostic Laboratory (Ames, IA) for vitamin analysis. Vitamin A and E analysis was conducted using high-pressure liquid chromatography as described in Greiner et al. (2022). Vitamin D was analyzed via liquid chromatography-mass spectrometry–mass spectrometry using Iowa State University Veterinarian Diagnostic Laboratory protocol number 9.7841. Vitamins are reported herein as their general names; however, vitamin A was analyzed for retinol in the serum, retinyl palmitate was converted to retinol for measurement in the liver, vitamin D was analyzed for the 25-hydroxy D_3_ in the serum, and vitamin E was analyzed for alpha-tocopherol in the serum and liver.

### Enzyme-Link Immunoassay for Immunoglobulins

Immunoglobulins (Ig) G and M in pig plasma were analyzed via enzyme-link immunoassay. Polystyrene plates (CAT: 15041; ThermoFisher Scientific) were washed four times before starting the assay with a wash solution (phosphate buffer saline with 0.5% tween, room temperature [roughly 20 °C], pH 7.4). Plates received 100 μL of the primary antibody (IgG CAT: AAI41; IgM CAT: AAI48; Bio-Rad Laboratories, Inc., Hercules, CA, USA) and 100 μL coating buffer (CAT: CB01100; ThermoFisher Scientific) per well, and were incubated for 1 h at room temperature. Plates were then washed three times with a wash buffer, and a blocking agent (100 μL; CAT: DS98200, ThermoFisher Scientific) was added to each well. The plate was incubated overnight at room temperature and washed three times with the wash buffer the next day. A standard curve was included on every plate using a pig IgG (CAT: I4381-10MG; Millipore Sigma; Burlington, MA) or pig IgM (CAT: 5276-6504; Bio-Rad Laboratories, Inc.), respective to the assay being conducted. The native pig antibodies were diluted to 1000 ng/mL, and then serial diluted to 333.33, 111.11, 37, 12, 4, and 1 ng/mL to create a standard curve. A sample of the diluent (**PBS**) was also included as a blank. Samples were diluted so that absorbance would be captured by the standard curve. The diluted sample (1:500,000 diluted sample for IgG or 100 μL of 1:250,000 diluted sample for IgM) were plated (100 μL) in duplicate, left to incubate for 1 h at room temperature, and were then washed three times with wash buffer. A secondary antibody (100 μL; IgG CAT: AA141P; IgM CAT: AAI48P Bio-Rad Laboratories, Inc.) was plated, and the plate was covered with foil and incubated at room temperature for 1 h, then washed three times. Plates were coated with 3,3ʹ5,5ʹ-tetramethylbenzidine chromogen solution (100 μL CAT: 002023, ThermoFisher Scientific), covered with foil, and incubated at room temperature for 20 min. A stop solution (100 μL; 0.16 M sulfuric acid) was added to stop the reaction. Plates were read (Cytation 5 Hybrid Multi-Mode Reader, Biotek Instruments Inc., Winooski, VT) at 450 nm. An intra-assay coefficient of variation of under 8% between duplicates within a plate was deemed acceptable. Inter-assay coefficient of variation was calculated to be 31.84% and 39.77% for IgG and IgM, respectively.

### Vitamin A Metabolism Polymerase Chain Reaction

Total RNA from jejunal samples was isolated via the Qiagen RNeasy Mini Kit (Qiagen, Germantown, MD) used according to the manufacturer’s instructions, specifically using the protocol listed for “Purification of Total RNA from Animal Tissues.” RNA isolation was conducted at 20 °C. Approximately 30 mg of jejunal tissue was homogenized using the Qiagen TissueLyzer II (Qiagen) with 600 μL Buffer RLT with 1% beta-mercaptoethanol. After homogenization, the lysate was centrifuged at 3 min at 17,000 × *g*. Ethanol (70%, 450 μL) was added to the isolated supernatant and mixed thoroughly. All samples were centrifuged using an accuSpin Micro 17R centrifuge (Fisher Scientific, Waltham, PA) at room temperature (approximately 20 °C) for RNA isolation. Up to 700 μL of the isolate and ethanol mixture was moved to a spin column in a 2-mL collection tube (provided with the kit) and centrifuged for 1 min at 12,000 × *g*. Buffer RPE was added twice at 500 μL. After each buffer addition, the sample was centrifuged, first for 1 min at 15,000 × *g* then at 15,000 × *g* for 2 min. The spin column was placed in a new 2-mL collection tube, then centrifuged at 17,000 × *g* for 1 min. The spin column was then placed in a 1.5-mL collection tube, and 50 μL RNAse-free water was added directly to the membrane, then centrifuged for 1 min at 15,000 × *g* to collect the elution. The concentration of RNA was quantified via a spectrophotometer (ND-100; Nanodrop Technologies, Inc., Rockland, DE). All samples had a 260:280 ratio greater than 1.8.

Complimentary DNA (cDNA) was transcribed via the QuantiTect Reverse Transcription Kit (Qiagen, Hilden, Germany). The kit was used according to the manufacturer’s instructions. RNA was first diluted to obtain 0.8 µg RNA. A reaction of 2 μL gDNA wipeout buffer, 8 μL diluted sample RNA, and 4 μL RNAse-free water was incubated at 42 °C for 2 min. After the incubation, a reverse-transcriptase master mix (1 μL Quantiscript Reverse Transcriptase, 4 μL Quantiscript RT Buffer, and 1 μL Primer Mix) was added. After the reverse-transcriptase master mix was added, the reaction was then incubated at 42 °C for 15 min, then 3 min at 95 °C. All cDNA samples were then diluted 10-fold with nuclease-free water to be used for real-time quantitative polymerase chain reaction (**PCR**).

To perform PCR, iQ SYBR Green Supermix (Bio-Rad Laboratories, Inc.) was used. All reactions totaled 20 µL, which consisted of 10 µL of SYBR Green Supermix, 5 µL of nuclease-free water, 3 µL of cDNA, 1 µL of the forward primer, and 1 µL reverse primer. Additionally, each 96-well plate contained a no-reverse transcriptase negative control and a pooled cDNA reference sample. Real-time PCR Detection System (iQ5; Bio-Rad Laboratories Inc.) was used to quantify the fluorescence of SYBR Green. Cycling conditions and gene-specific primers (diluted to 10 µM with nuclease-free water) are documented in Table 2. Genes include retinol binding protein 2 (RBP2), Alcohol dehydrogenase class 1 (ADH1), lecithin retinol acyltransferase (LRAT), and beta-carotene oxygenase 1 (BCO1). Glyceraldehyde-3-phosphate dehydrogenase (GAPDH) was included as an endogenous reference.

**Table 2.** Primer sequences and annealing temperatures used for measuring mRNA abundance of proteins involved in vitamin A metabolism in nursery pigs

Gene	Oligo name	Accession number	Sequence direction	Primer sequence	Annealing temperature (°C)	Citation
Retinol binding protein 2	RBP	NM_214451	Sense	AGGAGAACCGAGGCTGGAA	57	Arce et al., 2014
			Antisense	ACACCTGGTCACCGCATGT		
Alcohol dehydrogenase class 1	ADH1	NM001243939	Sense	ACCCCCGAAGGCCTATGAAGT	60	Ayuso et al., 2015
			Antisense	CTGGGGAACAAAGAGTGGGATGA		
Lecithin retinol acyltransferase phosphatidylcholine-retinol O-acyltransferase	LRAT	NM00124492	Sense	CTCAAGAAGAAGGCGCTGCTCAA	60	Ayuso et al., 2015
			Antisense	ATTATCTTCACATTCTCACAAAA		
Beta-carotene oxygenase 1	BCO1	NM_001136512.1	Sense	GCCAGAGTGACAGGCAAGAT	60	Designed using NCBI Primer Blast
			Antisense	TTGTACTTGGTCTCGCCCAC		
Glyceraldehyde-3-phosphate dehydrogenase	GAPDH	AF017079	Sense	GAAGGTCGGAGTGAACGGAT	55	Shang et al., 2019
			Antisense	CATGGGTAGAATCATACTGGAACA		

### Feed Chemical Analysis

A feed sample (at least 300 g) was collected after each batch of feed was manufactured at the Iowa State University Swine Nutrition Research Farm (Ames, IA) and stored at −20 °C until further analysis. A composite sample (200 g) of each phase was made from equal amounts of each batch and utilized for feed chemical analysis. Feed samples were ground using a Wiley Mill (Variable Speed Digital ED-5 Wiley Mill; Thomas Scientific, Swedesboro, NJ) to obtain a particle size of 1 mm. Proximate analysis of the ground composite sample was performed in duplicate to obtain gross energy using an isoperibolic bomb calorimeter (model 6200; Parr Instrument Co., Moline, IL) and acid hydrolyzed ether extract via a SoxCap hydrolyzer (model SC 247) and a Soxtec fat extractor (model 255; Foss, Eden Prairie, MN; method 968; AOAC, 2007). Standards used for calibration gross energy analysis were achieved using benzoic acid (Parr Instrument Co.; 6,318 kcal GE/kg).

All feed samples were analyzed in triplicate for vitamin A as retinyl acetate at Iowa State University Veterinary Diagnostic Laboratory via a protocol that was developed in-house (protocol #9.2020, unpublished).

### Statistical Analysis

All data were analyzed using the GLIMMIX procedure in SAS 9.4 (SAS Inst., Cary, NC). Pen was the experimental unit with fixed effects of diet and time and a random effect of pen. Repeated measures were utilized for growth performance and blood analysis. The main effects included diet, sex, time, and their interactions were appropriate. A spatial power covariate was included to account for an unequal amount of days in each phase. Means were reported as LSMeans using a Tukey adjustment. *P*-values ≤ 0.05 were considered significant, while 0.05 < *P*-values ≤ 0.10 were considered a tendency.

Outliers were determined by a measurement being more than three standard deviations from the mean, with that sample not contributing to the calculation of the mean. Pens were removed from growth performance analysis if they were deemed an outlier for more than one measured parameter (two pens total). Pigs were removed from PCR analysis if there was more than one outlier for the analyzed mRNA abundance (three pigs removed from analysis). Data were log-transformed for normality and back-transformed for reporting herein. Pigs were removed if both IgM and IgG were three standard deviations from the mean for 1 day or if either IgM or IgG were three standard deviations from the mean for both days samples were taken (seven pigs removed from analysis).

Animals that contributed to the baseline collection for liver vitamin A and PCR work were analyzed via the GLIMMIX procedure in SAS for a least square mean and associated standard error of means.

## Results

The analyzed vitamin A (Table 3) was not consistent, and there was a wide range of vitamin A recovered across samples. Standard deviations range from <2,907 IU/kg complete feed to 15,697.7 IU/kg complete feed. Analysis was done with a small amount of feed from a large batch, which could lead to the inconsistencies in the analysis. It is assumed that the pig will consume enough feed over the course of the study to have ingested the anticipated amount of vitamin A.

**Table 3.** Dietary vitamin A analysis across three nursery phases with no added fat, 3% added saturated fat, or 3% unsaturated fat with either a vitamin A (VA) or beta-carotene (BC) supplementation

	0% fat		3% saturated fat		3% unsaturated fat	
Parameter^1^	VA	BC^2^	VA	BC^2^	VA	BC^2^
Expected vitamin A	11,656	7,656	11,656	7,656	11,656	7,656
Analyzed vitamin A phase 1	16,860.5	<2,907.0	<2,907.0	9,011.6	9,593.0	6,395.3
Analyzed vitamin A phase 2	2,907.0	9,302.3	8,720.9	4,651.2	8,139.5	3,779.1
Analyzed vitamin A phase 3	12,209.3	3,779.1	3,779.1	10,174.4	19,186.0	3,197.7

^1^Vitamin A values reported in IU/kg of diet. Vitamin A was analyzed as retinyl acetate by the Iowa State University Veterinarian Diagnostic Laboratory.

^2^The expected levels on vitamin A in the feed when supplemented with beta-carotene is lower than anticipated vitamin A supplied to the pig by the diet with beta-carotene supplementation. This is because beta-carotene needs to be biologically cleaved to vitamin A. Thus the expected vitamin A levels in the feed are lower than what the pig will be exposed to, as they will convert beta-carotene to vitamin A.

**Figure 1.**
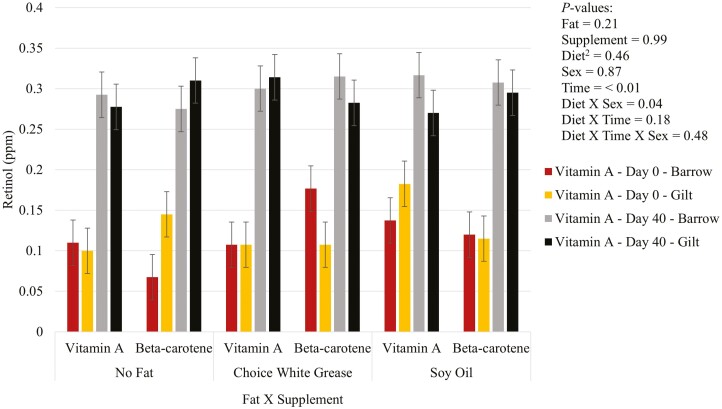
Added dietary fat and supplementation of vitamin A or beta-carotene on vitamin A^1^ in plasma of nursery pigs. ^1^Vitamin A is measured through the metabolite retinol in the plasma. ^2^Diet = fat × supplement.

**Figure 2.**
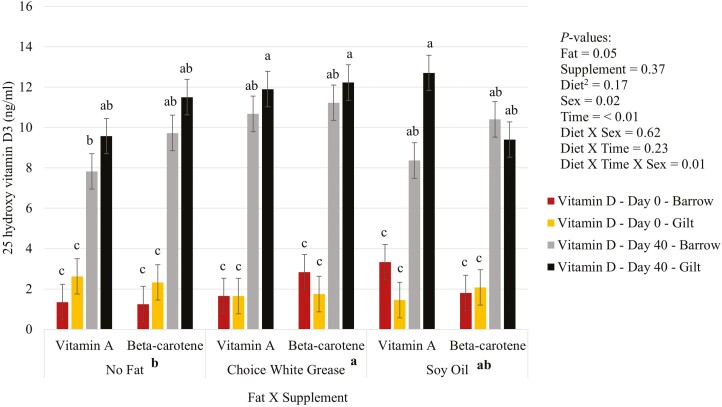
Added dietary fat and supplementation of vitamin A or beta-carotene on vitamin D^1^ in plasma of nursery pigs. ^1^Vitamin D measured through the metabolite 25 hydroxy vitamin D3 in the plasma. ^2^Diet = fat × supplement. ^a,b,c^Statistical significance.

**Figure 3.**
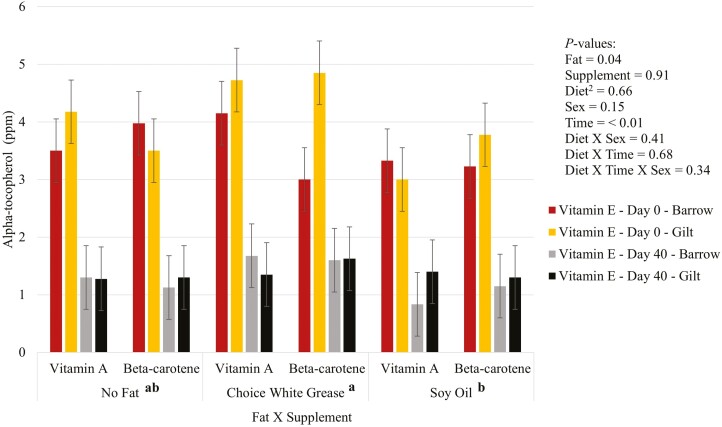
Added dietary fat and supplementation of vitamin A or beta-carotene on vitamin E^1^ in plasma of nursery pigs. ^1^Vitamin E was measured as the metabolite alpha-tocopherol in the plasma. ^2^Diet = fat × supplement. ^a,b^Statistical significances.

There was no effect (*P* > 0.05) of the fat by supplementation interaction on vitamins A (Figure 1), D (Figure 2), and E (Figure 3) in plasma. For vitamin D, There was a three-way interaction (*P* = 0.01) between diet, sex, and time, where gilts fed diets with choice white grease with vitamin A and beta-carotene, or gilts fed diets with soy oil and supplemented with vitamin A had higher plasma vitamin D than barrows that had no added fat diets and were supplemented with vitamin A. There was an effect (*P* < 0.05) of fat on both vitamins D and E. Pigs fed choice white grease diets had higher plasma vitamin D than diets that did not have added fat (averages of 6.74 vs. 5.77 ng/mL, respectively), and choice white grease diets led to higher plasma vitamin E than diets with soy oil (averages of 2.87 vs. 2.25 ppm, respectively). Additionally, gilts had (*P* = 0.02) a higher plasma vitamin D over barrows (6.60 vs. 5.87 ng/mL, respectively). There was a fat by supplement by sex interaction (*P* = 0.04) for plasma vitamin A, but overall, there was no difference (*P* > 0.05) in plasma vitamin A between diets at the end of the study. There was significantly (*P = *0.04; Table 4) higher hepatic vitamin A in pigs supplemented with vitamin A compared to pigs that were supplemented with beta-carotene (19.89 vs. 15.56 ppm, respectively).

**Table 4.** Added dietary fat and supplementation of vitamin A (VA) or beta-carotene (BC) on vitamins A and E concentration in the liver of nursery pigs at the end of the study (day 40)

			No fat				Choice white grease				Soy oil									
			VA		BC		VA		BC		VA		BC			*P*-values^1^^,^^2^				
	Baseline^3^	SEM	G^4^	B^5^	G	B	G	B	G	B	G	B	G	B	SEM	F	A	D	S	D × S
Vitamin A, ppm	29	3	20	24	15	15	23	19	15	16	21	13	14	19	3.4	—	*^6^	—	—	—
Vitamin E, ppm	6.1	1.1	3.0	3.3	3.9	3.5	3.6	4.0	3.2	3.9	3.9	1.6	3.1	3.2	0.76	—	—	—	—	—

^1^F = fat, A = supplement, D = fat × supplement, S = sex, T = time.

^2^—, not significant.

^3^Baseline pigs mean reported with the error term, not included in the overall statistical model.

^4^Gilt.

^5^Barrow.

^6^Pigs that were supplemented with vitamin A had higher (*P* = 0.04) hepatic vitamin A levels than pigs supplemented with beta-carotene.

The mean BW of pigs at the end of the study was 24.47 ± 0.06 kg. There was a main effect of fat (*P* < 0.05) for BW and average daily gain (**ADG**) when analyzed by repeated measures (Table 5) in which pigs that were fed diets with added soy oil had better growth performance than pigs with no added fat to their diets (BW, 25.97 vs. 22.22 kg, respectively; ADG, 0.65 vs. 0.59 kg, respectively). There was a tendency (*P* = 0.06) for pigs to have a higher average daily feed intake (**ADFI**) when supplemented with soy oil than pigs with no added fat. Additionally, there was a fat by supplementation interaction (*P* = 0.03) for BW when analyzed by repeated measures (Table 6) in which pigs supplemented with soy oil and beta-carotene had a higher BW than pigs supplemented with no added fat and beta-carotene (26.83 vs. 20.91 kg, respectively), although this was not observed across phases. Pigs had (*P* = 0.05) a heavier BW when supplemented with soy oil than pigs with no added fat (Table 7). However, there was no difference (*P* > 0.05; Table 7) for fat by supplementation in the final BW of the pigs at the end of the study. No differences (*P* > 0.05) were observed for ADFI or ADG (Table 7). There was an improved overall gain:feed (*P* = 0.02) when pigs were supplemented with choice white grease over no added fat (0.72 vs. 0.69, respectively).

**Table 5.** Main effects of added dietary fat or supplementation (sup) of vitamin A (VA) or beta-carotene (BC) on growth performance in nursery pigs by phase

	Fat					Supplementation				*P*-values^1^	
	No fat	Choice white grease	Soy oil	SEM	*P*-value	VA	BC	SEM	*P*-value	Fat × sup	Fat × sup × day
ADFI, kg				0.030	0.06			0.024	0.19	0.79	0.83
Days 0 to 7	0.18	0.20	0.20			0.19	0.20				
Days 8 to 21	0.47	0.50	0.57			0.50	0.52				
Days 22 to 40	0.93	0.93	0.98			0.92	0.97				
ADG, kg				0.022	0.02			0.019	0.23	0.79	0.96
Days 0 to 7	0.17	0.19	0.19			0.19	0.19				
Days 8 to 21	0.37	0.40	0.44			0.40	0.41				
Days 22 to 40	0.59	0.61	0.65			0.60	0.64				
GF				0.043	0.71			0.035	0.79	0.65	0.150
Days 0 to 7	0.98	1.00	0.96			1.00	0.96				
Days 8 to 21	0.77	0.80	0.79			0.79	0.79				
Days 22 to 40	0.63	0.66	0.66			0.65	0.66				
BW, kg				0.643	<0.01			0.519	0.91	0.03	0.79
BW0	5.65	5.70	6.07			5.82	5.81				
BW7	6.86	7.05	7.43			7.12	7.11				
BW21	11.51	12.69	13.63			12.74	12.48				
BW40	22.22	24.35	25.97			24.13	24.24				

^1^There was a significant day effect (*P* < 0.0001) for all parameters.

**Table 6.** Effects of added dietary fat and supplementation (sup) of vitamin A (VA) or beta-carotene (BC) on growth performance in nursery pigs by phase

Fat	No fat		Choice white grease		Soy oil			*P*-values^1^	
Sup	VA	BC	VA	BC	VA	BC	SEM	Fat × Sup	Phase × Fat × Sup
ADFI, kg							0.043	0.79	0.83
Days 0 to 7	0.18	0.17	0.18	0.23	0.20	0.20			
Days 8 to 21	0.48	0.46	0.49	0.51	0.54	0.59			
Days 22 to 40	0.90	0.95	0.92	0.94	0.95	1.02			
ADG, kg							0.034	0.79	0.96
Days 0 to 7	0.18	0.16	0.19	0.20	0.19	0.20			
Days 8 to 21	0.37	0.36	0.38	0.42	0.44	0.44			
Days 22 to 40	0.57	0.60	0.60	0.63	0.62	0.68			
GF							0.063	0.65	0.15
Days 0 to 7	0.99	0.97	1.11	0.90	0.91	1.01			
Days 8 to 21	0.76	0.79	0.78	0.83	0.82	0.76			
Days 22 to 40	0.63	0.63	0.65	0.68	0.65	0.67			
BW, kg							0.940	0.03	0.79
BW0	5.91	5.40	5.66	5.75	5.88	6.27			
BW7	7.15	6.58	6.99	7.11	7.22	7.65			
BW21	12.48	10.53	12.34	13.03	13.39	13.87			
BW40	23.52	20.91	23.74	24.97	25.12	26.83			

^1^Phase was significant (*P* < 0.001) for all parameters.

**Table 7.** Overall effects of added dietary fat and supplementation (sup) of vitamin A (VA) or beta-carotene (BC) on growth performance in nursery pigs

	Fat					Supplementation				*P*-value
	No fat	Choice white grease	Soy oil	SEM	*P*-value	VA	BC	SEM	*P*-value	Fat × Sup
BW, kg										
Day 0	5.65	5.70	6.07	0.242	0.41	5.82	5.81	0.195	0.97	0.41
Day 40	22.22	24.35	25.97	1.064	0.05	24.13	24.24	0.859	0.93	0.30
Overall, days 0 to 40										
ADFI, kg	0.63	0.65	0.70	0.030	0.31	0.64	0.68	0.024	0.36	0.86
ADG, kg	0.43	0.47	0.50	0.021	0.15	0.45	0.48	0.017	0.31	0.89
G:F	0.69^b^	0.72^a^	0.71^ab^	0.008	0.02	0.70	0.71	0.006	0.49	0.32

^a,b^Statistical differences.

Neither sex nor diet altered (*P* > 0.05; Table 8) Ig levels in the plasma for either IgG or IgM. There was a significant across time for both Ig (*P* = 0.02), where IgG levels were higher at the start (22.14 vs. 14.00 ng/mL, respectively) and IgM levels (*P* = < 0.01) were higher at the end of the study (4.32 vs. 10.68 ng/mL, respectively). The change in the amount of immunoglubins across time indicates that the pig’s immune system is developing; however, the change is not influenced by dietary treatment.

**Table 8.** Added dietary fat and supplementation of vitamin A (VA) or beta-carotene (BC) on immunoglobulins (Ig) G and M in nursery pigs

	No fat				Choice white grease				Soy oil									
	VA		BC		VA		BC		VA		BC			*P*-values^1^^,^^2^				
	G^3^	B^4^	G	B	G	B	G	B	G	B	G	B	SEM	F	A	D	S	T
IgG, ng/mL^5^													9.035	—	—	—	—	0.02
Day 0	33.66	14.22	32.64	19.28	19.59	19.55	18.14	16.85	18.31	17.43	12.23	43.86						
Day 40	16.56	14.27	13.63	17.90	14.78	10.66	14.21	15.84	16.82	9.12	10.19	14.14						
IgM, ng/mL^6^													2.854	—	—	—	—	**
Day 0	6.04	4.20	5.05	3.93	3.39	4.13	4.67	5.65	2.63	2.93	1.96	7.26						
Day 40	12.20	10.72	9.09	14.46	9.46	11.33	8.09	13.00	8.77	7.16	13.57	10.33						

^1^F = fat, A = supplement, D = fat × supplement, S = sex, T = time; no significant interactions were noted between fat × supplement × sex, fat × supplement × time, or fat × supplement × sex × time.

^2^—, not significant; **, <0.001.

^3^Gilt.

^4^Barrow.

^5^Samples diluted to 1:500,000 for IgG analysis.

^6^Samples diluted to 1:250,000 for IgM analysis.

There was a tendency (*P* = 0.08; Table 9) of fat by supplement for the ADH gene in which added choice white grease with a beta-carotene supplementation has the highest mRNA abundance and added soy oil with beta-carotene supplementation has the lowest mRNA abundance. No other differences (*P* > 0.05) were noted for other measured mRNA of genes across the dietary treatments.

**Table 9.** Added dietary fat and supplementation (supp) of vitamin A (VA) or beta-carotene (BC) on mRNA abundance of genes involved in vitamin A metabolism in nursery pigs

			No fat				Choice white grease				Soy oil									
			VA		BC		VA		BC		VA		BC			*P*-values^1^^,^^2^				
	Base^3^	SEM	G^4^	B^5^	G	B	G	B	G	B	G	B	G	B	SEM	F	A	D	S	D × S
Gene^6^																				
ADH	0.32	2.76	0.99	0.54	2.02	1.59	0.51	2.62	1.64	1.98	1.02	2.69	0.76	0.58	1.867	—	—	0.08	—	—
BCO1	0.20	1.93	4.77	5.71	23.80	7.01	5.64	10.45	7.17	1.55	5.69	12.53	4.58	11.20	2.064	—	—	—	—	—
LRAT	0.87	1.61	4.90	4.11	7.15	2.93	4.94	6.72	3.29	2.39	6.66	4.65	2.70	5.66	1.840	—	—	—	—	—
RBP	0.96	2.19	1.75	1.97	4.01	2.85	2.31	4.42	4.34	1.04	2.04	2.58	2.87	4.66	1.562	—	—	—	—	—

^1^F = fat, A = supplement, D = fat × supplement, S = sex.

^2^—, not significant.

^3^Baseline pigs mean reported with the error term, not included in the overall statistical model.

^4^Gilt.

^5^Barrow.

^6^ADH, alcohol dehydrogenase 1C (class 1); BCO1, beta-carotene oxygenase 1; LRAT, lecithin retinol acyltransferase (phosphatidylcholine-retinol O-acyltransferase); RBP, retinol binding protein 2.

## Discussion

The lack of difference in vitamin A levels in the plasma from the dietary treatments was expected as plasma vitamin A is highly regulated (Tanumihardjo, 2011). The overall increase in plasma vitamin A for all dietary treatments over the course of the study could indicate the pig’s need for vitamin A in the peripheral tissues increases with age. This makes sense as vitamin A plays a role in epithelium cell differentiation, cell growth, and immune function (Huang et al., 2018; Barbalho and Pescinini-Salzedas, 2019), which are all still developing in the young pig. There is also a chance that the amount of vitamin A in the complete feed is more than in sow milk, thus there was a general increase due to exposure. Although the additional supplementation of vitamin A did not result in an increase in plasma vitamin A. The increase in hepatic vitamin A with the vitamin A supplementation was anticipated as the body will store the excess vitamin A (19.89 vs. 15.56 ppm). The levels found herein for both plasma and liver were similar to those listed in Hinson et al. (2022): approximately 0.20 ppm in blood and roughly 20 ppm in the liver. Additionally, there was no difference in hepatic vitamin A regardless of the amount or type of fat added to the diet.

While the study had an initial interest in analyzing vitamin A, vitamins E and D were also analyzed to see if added fat had an effect on these fat-soluble vitamins. Vitamin D was higher in the diets with choice white grease compared to the no added fat, which was expected as vitamin D is a fat-soluble vitamin. Interestingly, choice white grease diets had the highest vitamin D levels in the plasma, which was unexpected as saturated fats tend to be harder to digest due to the lack of double bonds in the fatty acid chains (Powles et al., 1995). Interestingly, soy oil naturally contains a lot of vitamin E (Bieri and Evarts, 1974), yet plasma vitamin E was lowest in diets supplemented with soy oil. This could result from polyunsaturated fatty acids in the oil causing oxidation. Vitamin E is a potent antioxidant, and the lower levels could indicate that vitamin E is being utilized as an antioxidant in the animal, and this leads to the question of whether vitamin E supplementation is needed with the use of unsaturated fats in diets. Once more, both plasma vitamin E (~1.50 ppm) and liver vitamin E (~3.0 ppm) were similar to values reported in the industry (Hinson et al., 2022).

Adding fats to nursery diets can increase the palatability to feed and may encourage the young pig to eat more (Miller et al., 1991). Also, the addition of fat to the diet results in better absorption of carbohydrates and amino acids (Miller et al., 1991), which could result in better growth of pigs. Furthermore, adding fat to finishing pig diets has been shown to increase ADG and feed efficiency (De la Llata et al., 2001; Stephenson et al., 2016). The lack of overall differences observed herein for growth performance can be attributed to the fact that growing pigs use supplemental fat differently depending on age, as concluded by Miller et al. (1991). Previously reported benefits of fat being added to the diet are in grow-finish pigs, not nursery (De la Llata et al., 2001; Stephenson et al., 2016). One reason that nursery pigs may not see the same benefits as finishing pigs can be due to a smaller amount being consumed, as hypothesized in Miller et al. (1991). Although, there have also been both positive and negative effects on the growth of nursery pigs when fat is added to nursery diets, as summarized in Miller et al. (1991). It was observed in this study that fat tended increase feed intake over the phases of the study, which could be due to an increase in palatability from the added soy oil. Diets that did not receive added fat were lower in metabolizable energy (approximately 135 kcal/kg lower); however, the main objective of this study was focused on if the vitamin status of the pig could be influenced by added fat, the difference in metabolizable energy is not a concern herein, but could be an explanation for the numerical difference in final BW. The fat used in this study was to represent fat sources in the industry and to measure vitamin A, D, and E levels in nursery pigs to observe any differences with either no added fat, a saturated fat source, and an unsaturated fat source.

The analyzed amount of vitamin A in the feed was not consistent with the formulated amount. To a degree, this is expected as the amount of vitamin A included in the diet is minute in comparison to other ingredients. Additionally, the pig will consume more feed than the amount of feed that was analyzed. As the pig is consuming a large amount of feed, it is believed that over the course that the pig is fed the diet, they will consume feed to the formulated amount of the supplementation.

The genes chosen to have their abundance of mRNA evaluated were chosen to observe vitamin A metabolism in the small intestine. Beta-carotene oxygenase is responsible for cleaving beta-carotene into two retinal molecules. Alcohol dehydrogenase class 1 is responsible for converting retinal to retinol in the intestine, which is necessary to get vitamin A into circulation, and retinol binding protein 2 is responsible for moving vitamin A in the form of retinol through circulation. Lecithin retinol acyltransferase is used for the esterification of vitamin A. Glyceraldehyde-3-phosphate dehydrogenase (GAPDH) was included as an endogenous reference.

The abundance of mRNA from the jejunum presented a large error term for the ADH gene. To help ensure the readings were correct, the housekeeping gene, GAPDH, and cycle threshold values were checked, and the average cycle threshold value was 22.2. The large error term observed herein could be driving the observed fat by supplement tendency.

As previously discussed, the young pig is still developing appropriate mechanisms for digesting and absorbing fat in the intestine. This may also be true for the ADH enzyme. The variation seen particularly in ADH could be due to the maturation of enzymes in the gut of nursery pigs. It should be noted that the paper in which the sequence for ADH was found, Ayuso et al. (2015), showed a relatively large error term for ADH as well in younger pigs, although this paper looked at ADH expression in the liver, not jejunum. Furthermore, it was noted that most of the vitamin A metabolism mRNA abundance for genes was not significantly greater than a control unless the pig had been exposed to high levels for a long period of time (i.e., ~30 to ~160kg; Ayuso et al., 2015).

Studies targeting mRNA abundance for genes involved in vitamin A metabolism in the jejunum of nursery pigs should further explore the maturation of this gene. As previously mentioned, the age of the pig affects the ability to utilize supplemental fat (Miller et al., 1991), so differences in fat types and their inclusion may have different effects in an older pig. Thus, other options should be explored to try and alter the abundance of mRNA for genes involved in vitamin A metabolism in nursery pigs. Additionally, histology measurements of the intestine could help to provide insight into the cells extracted to measure mRNA abundance.

This study demonstrated that the use of choice white grease resulted in higher levels of circulating vitamins E and D than diets with soy oil and no added fat, respectively. Supplementing fat, vitamin A, or beta-carotene did not change the circulating levels of vitamin A but did increase hepatic stores. If vitamin A is supplemented up to 11,656 IU/kg complete feed, hepatic vitamin A stores can be increased. Also, added soy oil to diets tended to increase feed intake in nursery pigs, although it did not improve feed efficiency. Circulating IgG and IgM were not affected by supplemented fat, vitamin A, or beta-carotene. The abundance of mRNA for genes associated with vitamin A metabolism was unaffected either by supplemental fat, vitamin A, or beta-carotene, or by the was a large error term that could be interpreted as ongoing maturation in nursery pigs. The use of soy oil in nursery pig diets does increase feed intake but will result in lower circulating vitamin E levels.
